# Insights into physical activity and cardiovascular disease risk in young children: IDEFICS study

**DOI:** 10.1186/1741-7015-11-173

**Published:** 2013-07-30

**Authors:** Robert G McMurray

**Affiliations:** 1 Fetzer Hall, University of North Carolina, Chapel Hill, NC, USA

**Keywords:** Blood pressure, Lipids, Glucose intolerance, Body fat, Accelerometry, Cardiometabolic risk factors

## Abstract

The association between physical activity and cardiovascular disease risk factors in children has been the focus of research for over two decades. The majority of this research has focused on children over 10 years of age with little information on very young children. The data recently published in *BMC Medicine* by Jiménez-Pavón and colleagues suggest that adverse cardiovascular disease (CVD) risk profiles, as indicated by a clustered risk score for the metabolic syndrome, are evident in very young children (two to six years of age), but differ between the sexes. The authors evaluated the relationship of CVD risk profiles and protective levels of moderate-to-vigorous physical activity (MVPA) and concluded that boys aged six years or younger needed >60 minutes of MVPA per day, whereas boys from six to nine years of age needed >80 minutes of MVPA per day; girls in either age group needed approximately 15 minutes less. Therefore, when clinicians recommend physical activity for children they should evaluate “at risk” children on a case-by-case basis rather than using generalized guidelines.

Please see related research: http://www.biomedcentral.com/1741-7015/11/172.

## Background

The importance of physical activity (PA) in preventing the development of cardiovascular disease (CVD) risk factors in children cannot be over stated. Numerous studies of children have shown that regular participation in PA can lower the risk of developing glucose intolerance, hyperlipidemia, elevated blood pressure and inflammation (see reviews [1,2]). However, the amount of PA necessary to improve CVD risk profiles remains elusive. Although recommendations vary, the consensus is that 60 minutes of daily moderate-to-vigorous PA (MVPA) is needed to improve health [3,4]; however, younger children may need more [5]. These recommendations may not be appropriate for all children, as numerous children meet these guidelines, but still have significant CVD risk [6]. Thus, the World Health Organization 2010 Guidelines [7] recommended 60 minutes of MVPA above habitual levels, to reduce CVD risk factors. The guidelines represent “best practice” and have not been truly evaluated in relation to health issues. The IDEFICS (‘Identification and prevention of Dietary - and lifestyle-induced health EFfects In Children and infantS’) study, presented in *BMC Medicine*, is one of the first to determine the amount of habitual PA needed to reduce the threat of developing an unhealthy CVD risk profile in children [8].

In addition, we know little about the progression of CVD risk factors in young children; at what age do children start to exhibit an unhealthy CVD profile? Previously, we noted that low levels of PA in 8- to 10-year olds, when children exhibit limited CVD risk, result in an increased risk of elevated CVD risk in late adolescence [9]. However, we were unable to determine if the risks are similar in girls and boys, and we did not have information on children less than eight years of age. The IDEFICS study helps provide information on this issue.

### Insights gained by the IDEFICS study

The IDEFICS study, authored by Jiménez-Pavón and associates, uses more than 3,000 two- to nine-year-old children from eight European countries to determine the relationships between objectively-monitored PA and a clustered CVD risk score. The clustered risk score was developed as part of the European Youth Heart Study as an alternative way of assessing metabolic syndrome for children and is derived by summing z-scores from each of the following measures: homeostatic model assessment of insulin resistance (HOMA), blood pressure, lipids and body mass index (BMI) or the sum of skinfolds. The size of the sample in the IDEFICS study allows the researchers to sub-divide the sample by sex into age groups (two- to six- and six- to nine-year-olds). Although the majority of the children were healthy, 15% of the sample was classified as having an unhealthy clustered CVD risk score. The weak inverse associations between PA levels and clustered CVD scores were as expected [1,10], weaker for the younger age group than the older age group. The relationships for young children (two to six years) varied by sex. No significant relationships were evident for the girls; however, those boys with the highest levels of PA (highest quintile) had reduced clustered CVD risk compared to the lowest quintiles. For older children (six to nine years) significant inverse relationships existed between total PA (or MVPA) and clustered CVD risk score. The PA recommendations to reduce CVD risk profile varied by age group and sex. Approximately 73 minutes/day of MVPA (moderate-to-vigorous) was necessary for young boys and 58 minutes/day for young girls. For the older children, approximately 85 minutes of MVPA was needed for boys and approximately 66 minutes for girls. These results suggest that greater amounts of PA are needed to influence the clustered CVD risk score as children age.

### Interpretations and future focal points

The IDEFICS study presents an examination of the CVD risk profiles and PA levels of young children. The study serves as a great initial step in our understanding of CVD risk in young children. The correlations between PA and CVD risk score suggest significant variability in the influence of PA on clustered CVD risk score, particularly in the young children. The weak associations could be related to the fact that children in the young age range have not had sufficient time to develop detrimental CVD profiles. In support, the data presented by the authors indicate that compared to the older children (six to nine years), the younger children (ages two to six years) had lower HOMA scores, blood pressures, total cholesterols, sum of skinfolds and clustered CVD risk scores. The weak associations may also have been produced by a combination of age- and sex-differences in the developmental trajectories, since there were many more differences in CVD risk factors between the sexes in the older age group than for the younger age group. This hypothesis is still in need of further clarification [11,12]. Finally, the weak association could also be related to considerable variations in PA levels for the same clustered CVD risk score.

Fatness is typically the most significant contributor to the clustered CVD risk score for children, and studies have documented the relationship between obesity and CVD risk factors [1,2,13-16]. Furthermore, there is an interaction between fatness and PA levels [17]. Thus, the IDEFICS study should further examine which factor, fatness or PA, is most salient.

Fatness may also reflect on cardiorespiratory fitness values [18]. The unit for fitness is ml per kg body weight per minute (mL/kg/min). Body weight includes both metabolically active tissue and fat mass. Fat mass contributes to energy requirements (of an activity), but not energy production. Thus, a fatter child usually has a lower maximal cardiorespiratory fitness value than a thinner child [18]. The shuttle run used in the IDEFICS study is an excellent means to assess aerobic or cardiorespiratory fitness (CRF) of children; using laps completed instead of computing CRFbased weight and laps completed may provide a better estimate of true aerobic fitness.

The authors suggest differing amounts of habitual PA are required to reduce CVD risk for younger versus older children, as well as for boys and girls; “one size does not fit all”. Older children required more PA and boys required more than girls. However, some highly active children can exhibit an unhealthy CVD risk profile and the results of the study do not attempt to clarify the amount of PA over-and-above habitual levels needed to reduce the risk profile [1,7]. Differences in PA levels between boys and girls have been previously noted [13], but no attempts have been made to relate PA to CVD risk in very young children. Since the physiological characteristics of very young boys and girls are similar, this topic is another area for future study.

## Conclusion

The results of the IDEFICS study presented by Jiménez-Pavón and colleagues provides a good “first look” at the relationship between PA levels and CVD risk in young children. This is important because there are little data on children of this age. The results suggest that even in young children, less than six years of age, some evidence of the metabolic syndrome (clustered risk) is accruing. Therefore, early childhood prevention should be the focus of future interventions. The study also provides substantive data on PA requirements based on health outcomes and suggests the importance of age and sex when prescribing PA. Therefore, clinicians should avoid using generalized guidelines for PA and evaluate “at risk” children on a case-by-case basis. The IDEFICS study has a very rich data set and it is hoped the authors will continue to provide us with new information.

## Abbreviations

BMI: Body mass index; CRF: Cardiorespiratory fitness; CVD: Cardiovascular disease; HOMA: Homeostatic model assessment of insulin resistance; IDEFICS: Identification and prevention of dietary- and lifestyle-induced health effects in children and infants; MVPA: Moderate-to-vigorous physical activity; PA: Physical activity.

## Competing interests

The author reports no competing interests, financial or otherwise.

## Author’s information

Robert G. McMurray is a Professor Emeritus from the Departments of Exercise and Sport Science and Nutrition, University of North Carolina. He has investigated cardiovascular disease risk factors and exercise in children for over two decades.

## Pre-publication history

The pre-publication history for this paper can be accessed here:

http://www.biomedcentral.com/1741-7015/11/173/prepub

## References

[B1] McMurray RG Ondrak KS Cardiometabolic risk factors in children: the importance of physical activity Am J Lifestyle Med 2013 [Epub ahead of print.]

[B2] Andersen LB Riddoch C Kriemler S Hills A Physical activity and cardiovascular risk factors in children Br J Sports Med 2011 45 871 876 10.1136/bjsports-2011-090333 21791456

[B3] Fulton JE Garg M Galuska DA Rattay KT Caspersen CJ Public health and clinical recommendations for physical activity and physical fitness: special focus on overweight youth Sports Med 2004 34 581 599 10.2165/00007256-200434090-00003 15294008

[B4] Colley RC Janssen I Tremblay MS Daily step target to measure adherence to physical activity guidelines in children Med Sci Sports Exerc 2012 44 977 982 10.1249/MSS.0b013e31823f23b1 22051570

[B5] Tremblay MS Leblanc AG Carson V Choquette L Connor Gorber S Dillman C Duggan M Gordon MJ Hicks A Janssen I Kho ME Latimer-Cheung AE Leblanc C Murumets K Okely AD Reilly JJ Spence JC Stearns JA Timmons BW Canadian physical activity guidelines for the early years (aged 0–4 years) Appl Physiol Nutr Metab 2012 37 345 356 10.1139/h2012-018 22448608

[B6] Andersen LB Harro M Sardinha LB Froberg K Ekelund U Brage S Anderssen SA Physical activity and clustered cardiovascular risk in children: a cross-sectional study (The European Youth Heart Study) Lancet 2006 368 299 304 10.1016/S0140-6736(06)69075-2 16860699

[B7] World Health Organization Global Recommendations on Physical Activity for Health 2010 Geneva, Switzerland: World Health Organization 26180873

[B8] Jimenez-Pavon D Konstabel K Bergman Ahrens W Pohlabeln H Hadjigeorgiou C Siani A Iacoviello L Molnár D De Henauw S Pitsiladis Y Moreno LA Physical activity and clustered cardiovascular disease risk factors in young children: a cross-sectional study (The IDEFICS study) BMC Med 2013 11 172 10.1186/1741-7015-11-172 23899208 PMC3728104

[B9] McMurray RG Bangdiwala SI Harrell JS Amorim LD Adolescents with metabolic syndrome have a history of low aerobic fitness and physical activity levels Dyn Med 2008 7 5 10.1186/1476-5918-7-5 18394155 PMC2358885

[B10] McMurray RG Andersen LB The influence of exercise on metabolic syndrome in youth: a review Am J Lifestyle Med 2010 4 176 186 10.1177/1559827609351234

[B11] Bugge A El-Naaman B McMurray RG Froberg K Andersen LB Tracking of clustered cardiovascular disease risk factors from childhood to adolescence Pediatr Res 2013 73 245 249 10.1038/pr.2012.158 23165452

[B12] Andersen LB Haraldsdottir J Tracking of cardiovascular disease risk factors including maximal oxygen uptake and physical activity from late teenage to adulthood. An 8-year follow-up study J Intern Med 1993 234 309 315 10.1111/j.1365-2796.1993.tb00748.x 8354982

[B13] Jago R Drews KL McMurray RG Thompson D Volpe SL Moe EL Jakicic JM Pham TH Bruecker S Blackshear TB Yin Z Fatness, fitness, and cardiometabolic risk factors among sixth-grade youth Med Sci Sports Exerc 2010 42 1502 1510 10.1249/MSS.0b013e3181d322c4 20139783 PMC2921216

[B14] Glowinska B Urban M Peczynska J Florys B Soluble adhesion molecules (sICAM-1, sVCAM-1) and selectins (sEselectin, sPselectin, sLselectin) levels in children and adolescents with obesity, hypertension, and diabetes Metabolism 2005 54 1020 1026 10.1016/j.metabol.2005.03.004 16092051

[B15] Ondrak KS McMurray RG Harrell JS The influence of aerobic power and percent body fat on cardiovascular disease risk in youth J Adolesc Health 2007 41 146 152 10.1016/j.jadohealth.2007.03.008 17659218

[B16] Reinehr T Kiess W de Sousa G Stoffel-Wagner B Wunsch R *Intima media* thickness in childhood obesity: relations to inflammatory marker, glucose metabolism, and blood pressure Metabolism 2006 55 113 118 10.1016/j.metabol.2005.07.016 16324929

[B17] Güvenç A Aslan A Açıkada C Objectively measured activity in 8-10-year-old Turkish children: relation to health-related fitness Pediatr Int 2013 [Epub ahead of print] 10.1111/ped.12119 23659616

[B18] McMurray RG Hosick PA Bugge A Importance of proper scaling of aerobic power when relating to cardiometabolic risk factors in children Ann Human Biol 2011 38 647 654 10.3109/03014460.2011.598561 21749316

